# Community versus individual risk of SARS-CoV-2 infection in two municipalities of Louisiana, USA: An assessment of Area Deprivation Index (ADI) paired with seroprevalence data over time

**DOI:** 10.1371/journal.pone.0260164

**Published:** 2021-11-30

**Authors:** Amy K. Feehan, Kara D. Denstel, Peter T. Katzmarzyk, Cruz Velasco, Jeffrey H. Burton, Eboni G. Price-Haywood, Leonardo Seoane

**Affiliations:** 1 Ochsner Clinic Foundation, New Orleans, LA, United States of America; 2 Ochsner Clinical School, The University of Queensland, New Orleans, LA, United States of America; 3 Pennington Biomedical Research Center, Louisiana State University, Baton Rouge, LA, United States of America; 4 Center for Outcomes and Health Services Research, New Orleans, LA, United States of America; 5 Louisiana State University Health Sciences Center-Shreveport, Shreveport, LA, United States of America; University of Miami, UNITED STATES

## Abstract

**Objective:**

Determine whether an individual is at greater risk of severe acute respiratory distress syndrome coronavirus 2 (SARS-CoV-2) infection because of their community or their individual risk factors.

**Study design and setting:**

4,752 records from two large prevalence studies in New Orleans and Baton Rouge, Louisiana were used to assess whether zip code tabulation areas (ZCTA)-level area deprivation index (ADI) or individual factors accounted for risk of infection. Logistic regression models assessed associations of individual-level demographic and socioeconomic factors and the zip code-level ADI with SARS-CoV-2 infection.

**Results:**

In the unadjusted model, there were increased odds of infection among participants residing in high versus low ADI (both cities) and high versus mid-level ADI (Baton Rouge only) zip codes. When individual-level covariates were included, the odds of infection remained higher only among Baton Rouge participants who resided in high versus mid-level ADI ZCTAs. Several individual factors contributed to infection risk. After adjustment for ADI, race and age (Baton Rouge) and race, marital status, household size, and comorbidities (New Orleans) were significant.

**Conclusions:**

While higher ADI was associated with higher risk of SARS-CoV-2 infection, individual-level participant characteristics accounted for a significant proportion of this association. Additionally, stage of the pandemic may affect individual risk factors for infection.

## Introduction

Disparities in severe acute respiratory syndrome coronavirus 2 (SARS-CoV-2) infection rates have been shown to be associated with community-level characteristics [1–4]. A Massachusetts, USA study found cities with greater proportions of Latino and Black residents had a higher SARS-CoV-2 prevalence. This finding was attenuated only for Latino populations when models adjusted for household size, percentage of food service workers, and percentage of foreign-born non-citizens [1]. A state and county-level analysis across the United States revealed that higher population percentages of Native American, Hispanic, and African American residents were associated with greater coronavirus disease (COVID-19) mortality [2]. Furthermore, race, which is a social construct and a proxy measure for social determinants of health, was the predominant predictor rather than population density or health status [2]. These differences in morbidity and mortality have been attributed to underlying disparities in occupation, education, transportation, and other markers of disadvantage [3]. A Louisiana study found a positive relationship between the Area Deprivation Index (ADI), a multidimensional measure of a community’s socioeconomic position, and SARS-CoV-2 infection in which there was a 45% higher risk of infection in the most deprived compared to the least deprived census tracts [5].

While population-level measures of the social determinants of health have been associated with area-level infection rates, the relative importance of individual- versus community-level risk factors on SARS-CoV-2 infection is not clear. An analysis of individual- versus community-level factors conducted in a cohort of 57,865 patients from an integrated healthcare system in New England revealed that independent of individual-level risk factors, SARS-CoV-2 infection rates were related to census tract-level socioeconomic characteristics including lower educational attainment and higher household crowding and occupancy, but not race [4].

The present study aimed to further address this gap in the literature by examining the associations between individual- and community-level factors and SARS-CoV-2 seropositivity. We used individual-level data from two seroprevalence studies conducted in New Orleans and Baton Rouge, Louisiana in combination with community-level ADI data generated using the 2018 5-year American Community Survey (ACS) [6]. We hypothesized that community ADI would be significantly associated with seropositivity, but that the association would be attenuated by individual-level variables.

## Methods

### Study variables and data collection

This study relies on 4,752 individual participant records collected with informed, verbal consent and documented electronically with an impartial witness in two seroprevalence studies conducted in New Orleans and Baton Rouge, Louisiana, USA [7, 8]. These seroprevalence studies were approved by the Ochsner Clinic Foundation (IRB #2020.163); detailed methods are available elsewhere [7, 8]. A representative sample of individuals provided informed consent before a nasopharyngeal swab, blood sample, and brief questionnaire. The questionnaire **(S1 Table)** administered in New Orleans contained fewer items than the one used in Baton Rouge, and the studies were conducted, respectively, May 9–15, 2020 after an initial wave of infections and a stay-at-home order, and July 15–31, 2020 after stay-at-home orders had expired, masks were mandated, and COVID-19 risk factors were clearer.

### ADI coefficient calculation

Because of population-level changes in Louisiana following Hurricane Katrina and to provide more proximate spatiotemporal matching between the area- and participant-level data, updated ADI coefficients were generated using data from the 2018 ACS five-year estimates for Louisiana zip code tabulation areas (ZCTA) [9]. ZCTAs are comprised of census blocks aggregated to create areal representations of U.S. Postal Service zip code service areas [10]. ADI coefficients were generated via a factor analysis of 17 concepts from the ACS that are indicators of poverty, housing, employment, and education and have been described previously [11]. These 17 measures were chosen [11] based on a factor analysis and their theorized relevance and relationship to mortality. Factor coefficients were originally calculated for various geography levels based on 1990 Census data [11], and the coefficients were updated for block groups using data from the 2000 Census [12]. Data were available from the 2018 ACS five-year estimates for all 515 Louisiana ZCTAs. We excluded 118 ZCTAs with missing data elements which led to incalculable ADIs, including four ZCTAs in which 25 study participants lived (70710, 70725, 70801, 70836). Therefore, a 1-factor solution factor analysis was performed for 397 Louisiana ZCTAs, and ADI was calculated using the calculated coefficients. A comparison of the ADI coefficients computed in this study with those from Singh [11] and Knighton et al. [12] are presented in **S2 Table**. The correlations between our computed coefficients and those of both Singh and Knighton et al. [11, 12] are r>0.99.

### Statistical analysis

Descriptive statistics were reported as means, standard deviations, and percentages as appropriate. Logistic regression models were estimated to assess associations of individual-level demographic and socioeconomic factors and community-level ADI with SARS-CoV-2 infection (0 = no infection; 1 = past (antibody positivity) or present (PCR positivity) infection). The Firth correction was used to address potential data sparseness effects. Analyses were performed separately for the Baton Rouge and New Orleans areas.

Continuous variables (ADI, BMI, age) were subjected to cubic splines fit with various number of knots to allow for non-linear association with probability of infection. Three knots were chosen for BMI and age (resulting in 4 groups) and 2 knots for ADI (resulting in 3 groups). Categories for these continuous factors are presented in Table 1.

**Table 1:** Associations between individual-level variables and SARS-CoV-2 infection in Baton Rouge and New Orleans.

Baton Rouge									New Orleans						
			Unadjusted ^a^		Model 1 ^b^		Model 2 ^c^					Unadjusted ^a^		Model 1 ^b^	
	N	Positive (%)	OR	95% CI	OR	95% CI	OR	95% CI		N	Positive (%)	OR	95% CI	OR	95% CI
Age group (cubic splines)	N	Positive (%)	OR	LL					Age group (cubic splines)	N	Positive (%)	OR	LL		
1: 18.1–37.2	523	52 (9.94)	-	-	-	-	-	-	1: 18.1–37.9	658	38 (5.78)	-	-	-	-
2: 37.2–48.9	522	28 (5.36)	**0.52**	**0.32, 0.82**	**0.47**	**0.28, 0.79**	**0.52**	**0.30, 0.89**	2: 37.9–50.9	655	46 (7.02)	1.23	0.79, 1.92	1.18	0.74, 1.90
3: 48.9–60.5	517	31 (6.00)	**0.58**	**0.36, 0.92**	0.66	0.38, 1.14	0.72	0.40, 1.25	3: 50.9–63.0	663	51 (7.69)	1.36	0.88, 2.10	1.33	0.81, 2.19
4: 60.5–91.4	525	13 (2.48)	**0.24**	**0.12, 0.42**	**0.35**	**0.16, 0.72**	**0.37**	**0.16, 0.80**	4: 63.0–99.2	655	46 (7.02)	1.23	0.79, 1.92	1.43	0.82, 2.49
Race									Race						
1: White, non-Hispanic	1480	65 (4.39)	-	-	-	-	-	-	1: White, non-Hispanic	1605	78 (4.86)	-	-	-	-
2: Black, non-Hispanic	506	55 (10.87)	**2.66**	**1.83, 3.85**	**2.20**	**1.46, 3.30**	**2.04**	**1.34, 3.10**	2: Black, non-Hispanic	823	90 (10.94)	**2.40**	**1.75, 3.29**	**2.31**	**1.58, 3.38**
3: Other	101	4 (3.96)	1	0.32, 2.38	0.76	0.25, 1.84	0.80	0.26, 1.94	3: Other	203	13 (6.40)	1.38	0.73, 2.43	1.36	0.72, 2.42
Sex									Sex						
1: Female	1330	82 (6.17)	-	-	-	-	-	-	1: Female	1667	122 (7.32)	-	-	-	-
2: Male	757	42 (5.55)	0.9	0.61, 1.31	1.00	0.67, 1.48	0.87	0.57, 1.33	2: Male	964	59 (6.12)	0.83	0.60, 1.14	0.93	0.66, 1.28
Comorbidities									Comorbidities						
1: Zero	1138	77 (6.77)	-	-	-	-	-	-	1: Zero	1441	91 (6.32)	-	-	-	-
2: One	539	24 (4.45)	0.65	0.40, 1.02	0.80	0.48, 1.30	0.82	0.48, 1.35	2: One	663	64 (9.65)	**1.59**	**1.14, 2.21**	1.26	0.87, 1.82
3: Two or more	410	23 (5.61)	0.83	0.51, 1.32	1.27	0.71, 2.24	1.26	0.69, 2.26	3: Two or more	527	26 (4.93)	0.78	0.49, 1.20	**0.55**	**0.32, 0.90**
Household size									Household size						
1: One person	339	22 (6.49)	-	-	-	-	-	-	1: One person	565	32 (5.66)	-	-	-	-
2: Two to three people	1208	47 (3.89)	0.58	0.35, 0.98	0.65	0.37, 1.16	0.56	0.31, 1.02	2: Two to three people	1525	111 (7.28)	1.29	0.88, 1.97	**1.81**	**1.16, 2.86**
3: Four or more people	540	55 (10.19)	1.61	0.98, 2.74	1.72	0.94, 3.22	1.42	0.76, 2.58	3: Four or more people	541	38 (7.02)	1.26	0.78, 2.04	**1.93**	**1.11, 3.37**
Marital Status									Marital Status						
1: Married/cohabiting	1236	60 (4.85)	-	-	-	-	-	-	1: Married/cohabiting	1368	82 (5.99)	-	-	-	-
2: Single	601	57 (9.48)	**2.05**	**1.41, 2.99**	1.42	0.88, 2.29	1.34	0.82, 2.18	2: Single	863	67 (7.76)	1.32	0.95, 1.84	**1.59**	**1.07, 2.33**
3: Divorced/widowed	250	7 (2.80)	0.60	0.26, 1.22	0.70	0.28, 1.51	0.65	0.26, 1.43	3: Divorced/widowed	400	32 (8.00)	1.38	0.89, 2.08	1.51	0.93, 2.40
BMI group (from cubic splines)	N	Positive (%)													
1: 16.4–24.3	524	31 (5.92)	-	-	-	-	-	-							
2: 24.3–28.0	525	30 (5.71)	0.96	0.58, 1.61	-	-	1.06	0.62, 1.83							
3: 28.0–32.9	517	27 (5.22)	0.88	0.52, 1.49	-	-	0.85	0.48, 1.51							
4: 32.9–70.6	521	36 (6.91)	1.18	0.72, 1.94	-	-	0.99	0.57, 1.72							
Work environment															
1: At home and on site	346	13 (3.76)	-	-	-	-	-	-							
2: Going in to work 100%	760	62 (8.16)	**2.21**	**1.25, 4.20**	-	-	1.64	0.90, 3.00							
3: Working from home 100%	454	22 (4.85)	1.29	0.65, 2.63	-	-	1.33	0.67, 2.62							
4: Unemployed/retired	527	27 (5.12)	1.36	0.71, 2.72	-	-	1.35	0.69, 2.64							
Job type															
1: Office	587	17 (2.90)	-	-	-	-	-	-							
2: Health Care	380	27 (7.11)	**2.54**	**1.38, 4.77**	-	-	**2.61**	**1.28, 5.31**							
3: Public Facing	330	29 (8.79)	**3.19**	**1.76, 5.97**	-	-	**2.38**	**1.20, 4.71**							
4: Other	257	24 (9.34)	**3.42**	**1.83, 6.53**	-	-	**2.73**	**1.35, 5.49**							
5: Unemployed/retired/unknown	533	27 (5.07)	1.77	0.97, 3.32	-	-	**2.11**	**1.11, 4.04**							

^a^ Bivariate associations between each variable and SARS-CoV-2 positivity.

^b^ ADI and all individual-level variables that were measured in both Baton Rouge and New Orleans (Age, race, sex, comorbidities, household size, and marital status).

^c^ ADI and all individual-level variables that were measured in only Baton Rouge (Age, race, sex, comorbidities, household size, and marital status, BMI, work environment, job type).

Three models were utilized to examine the association between ADI and odds of infection. The unadjusted model included three categories of ADI (low, mid and high) based on cubic splines analysis. Model 1 added covariates for the individual-level covariates measured in both municipalities (age, race, sex, comorbidities, household size, and marital status). Model 2 was fit with Baton Rouge data only and included all variables from Model 1 with additional individual-level variables collected during the Baton Rouge seroprevalence study (BMI, work environment, job type).

Similarly, a series of three models were used to examine the effects of individual-level risk factors on odds of infection. A series of bivariate logistic regression models were used to examine the association between each individual-level risk factor separately (unadjusted model). Model 1 included ADI and individual-level risk factors measured in both Baton Rouge and New Orleans, while Model 2 also included the individual-level covariates measured in Baton Rouge only.

A random effect for residential zip code was initially incorporated to account for potential variability in positivity rates across communities but was ultimately not included in the models due to low variability across zip codes and low or null response rates in several zip codes.

Results were reported as unadjusted and covariate-adjusted odds ratios and 95% confidence intervals. For odds ratios based on continuous measures, the unit change for calculations were specified in the tables.

Additional unadjusted analyses were carried out to assess associations between SARS-CoV-2 infection and each of the 17 individual concepts used to calculate ADI. Only one concept at a time was incorporated into the model due to strong inter-concept correlations. Analyses were performed using SAS/STAT 14.2.

## Results

**Fig 1:**
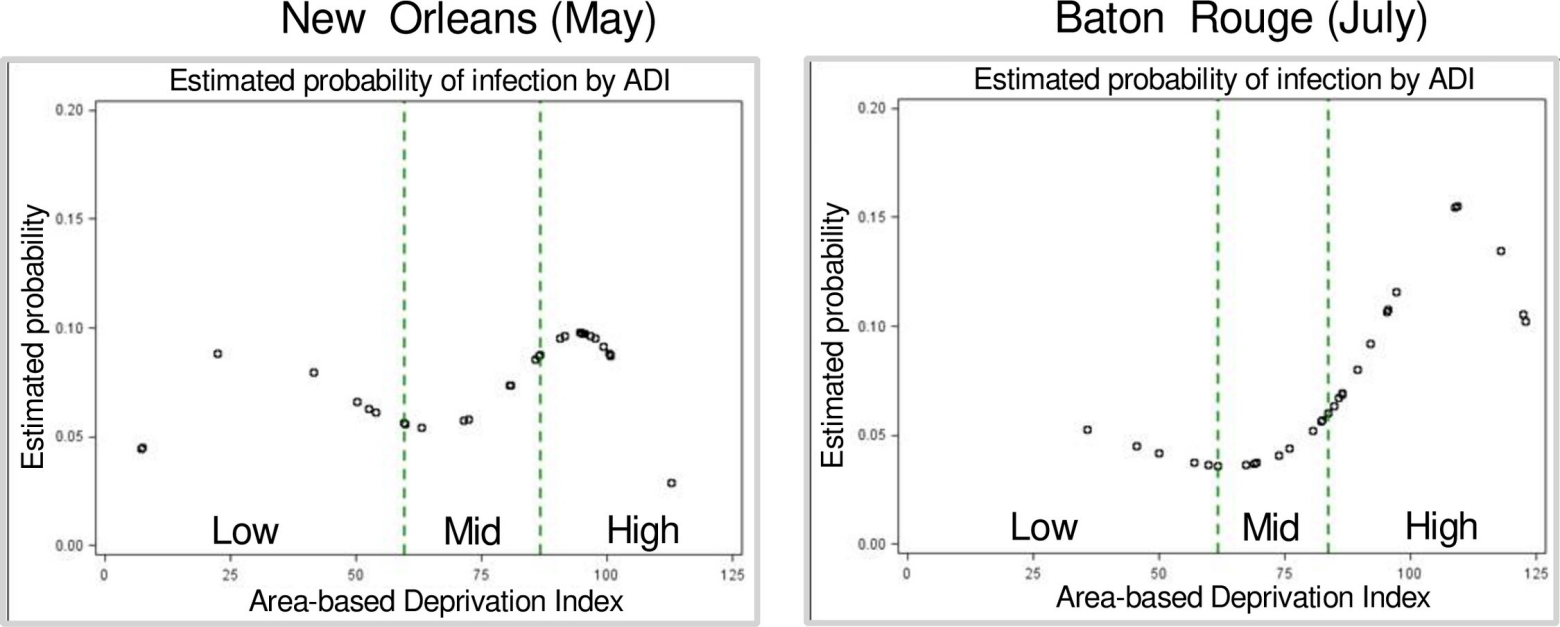
Association of area-based deprivation index (ADI) with probability of infection (splines, 2 knots). ADI ranges for low, mid, and high were (35.7–61.7), (61.7–83.6), and (83.6–112.9) for Baton Rouge and (7.3–59.5), (59.5–86.6), and (86.6–112.9) for New Orleans (denoted by dashed lines in the top panel).

**Fig 2:**
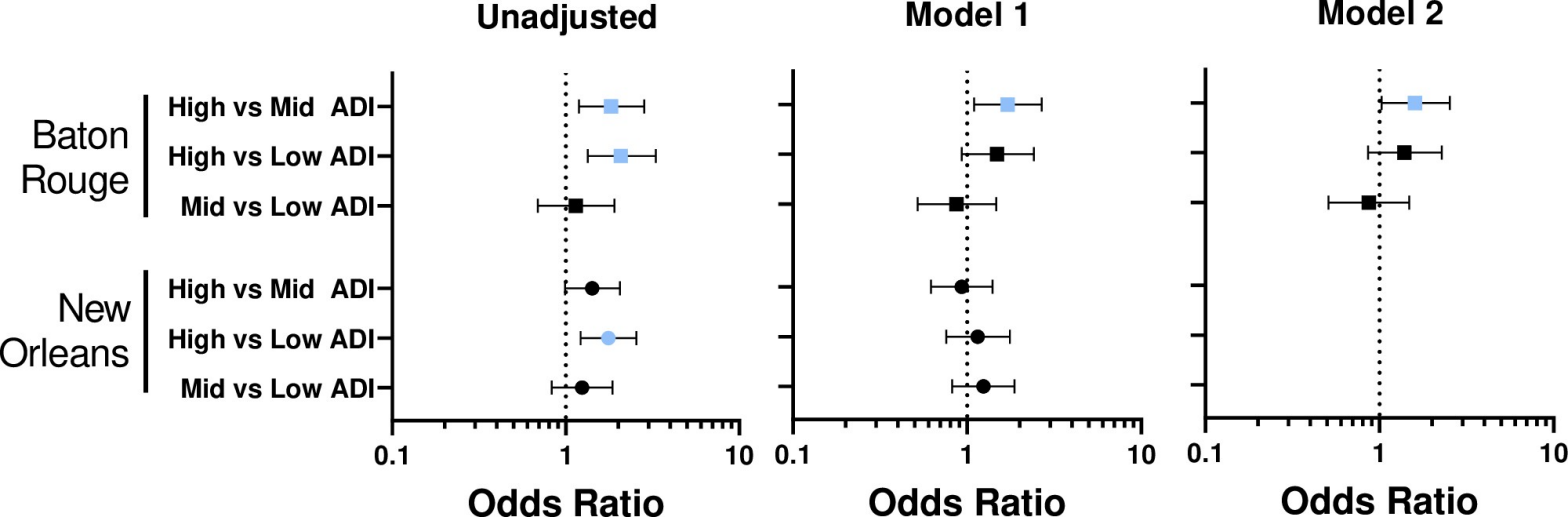
Association between ADI and SARS-CoV-2 infection in Baton Rouge and New Orleans. Results are presented as odds ratios; error bars represent 95% Confidence Intervals. The unadjusted model accounts for bivariate associations between each variable and COVID-19 positivity. Model 1 accounts for all individual-level variables that were measured in both Baton Rouge and New Orleans (Age, race, sex, comorbidities, household size, and marital status). Model 2 accounts for all individual-level variables that were measured in Baton Rouge (Age, race, sex, comorbidities, household size, and marital status, BMI, work environment, job type). BMI, work environment, and job type were not collected in New Orleans. ADI ranges for low, mid, and high were (35.7–61.7), (61.7–83.6), and (83.6–112.9) for Baton Rouge and (7.3–59.5), (59.5–86.6), and (86.6–112.9) for New Orleans.

In both samples, the risk of infection showed a curvilinear association with ADI (Fig 1). The dashed lines indicate the cut points used to define low (least deprived), mid and high (most deprived) ADI categories. Odds ratios comparing SARS-CoV-2 infection by ADI are presented in Fig 2. In the unadjusted model, there were greater odds of infection in both cities when comparing the high versus low ADI groups, and the odds of infection were also higher in the high versus mid-level ADI group in the Baton Rouge sample. When individual-level covariates were included, the odds of infection remained significantly higher in the high versus mid-level ADI group in Baton Rouge in both multivariable models (Model 1: OR = 1.71, 95% CI: 1.10–2.69; Model 2: OR = 1.60, 95% CI: 1.03–2.54). There were no significant associations once individual-level characteristics were included.

The odds of SARS-CoV-2 infection for the 17 ADI components are shown in S1 Fig. Higher community-level proportions of unemployment, poverty, single-parent households, and household crowding were associated with higher odds of infection in both New Orleans and Baton Rouge. Larger income disparity was also associated with greater odds of infection in New Orleans only. Conversely, higher income, monthly mortgage, home value, and white-collar employment rate were associated with lower odds of infection in both cities. Higher monthly rent in New Orleans and higher percentage with a high school diploma in Baton Rouge were also associated with lower odds of infection.

Table 1 presents associations between individual-level factors and the odds of SARS-CoV-2 infection. In Baton Rouge, older age groups (>37 y) had lower odds of infection compared to younger (ages 18–37 y) adults, while Black and single adults had higher odds of infection compared to White and married adults, respectively. Furthermore, people who were going into the workplace 100% had greater odds of infection compared those who were working from home part time. Across job types, people in healthcare, those with public-facing jobs, or other jobs had higher odds of infection compared to office workers. After adjustment for ADI and mutual adjustment for the individual-level risk factors in Model 1, the effects of age and race remained significant. After further adjustment for the additional factors in Model 2, the effects of age and race remained significant, and all job types had significantly elevated odds of infection compared to office work. In New Orleans, Black individuals and those with one comorbidity had elevated odds of infection compared to White individuals and those with zero comorbidities, respectively. After further adjustment for the ADI and mutual adjustment for other individual-level factors in Model 1, Black and single adults had elevated odds of infection compared to White and married adults, respectively. Further, there were elevated odds of infection among people with multiple people living in their household compared to living alone, and those with two or more comorbidities had lower odds of infection compared to those reporting zero comorbidities. A comparison of New Orleans and Baton Rouge Census data shows strong similarities in the populations (S3 Table).

## Discussion

This study found areas with the highest levels of community deprivation had the highest odds of SARS-CoV-2 infection, as reported in other U.S. regions [1–4, 13] and in Louisiana [14]. However, community deprivation was not significantly associated with odds of infection after accounting for individual-level variables, except in Baton Rouge where high versus middle ADI remained significantly associated with greater infection risk. Thus, individual-level variables were more consistently predictive of infection in our study compared to community-level ADI. However, the relationship between deprivation and SARS-CoV-2 infection is non-linear. In Baton Rouge, the curvilinear association indicates a dramatic increase in infection in the most deprived communities with the lesser deprived communities showing approximately equal probability of infection. In New Orleans, there is less variability across ADI groups, with the most deprived communities showing slightly elevated probability of infection compared to the lesser deprived. Lower variability in New Orleans could be caused by pandemic stage and heterogeneity of socioeconomic status and deprivation within the same ZCTAs, which may partly explain the attenuation of results in that sample.

Madhav et al. [5] examined the relationship between Louisiana census tract-level ADI and SARS-CoV-2 infection prevalence. They found that more deprived census tracts had 10% to 18% higher COVID-19 rates compared to the least deprived census tracts. Similarly, a longitudinal study [15] in seven U.S. states found that zip codes with higher ADI (i.e., more disadvantaged) had higher COVID-19 prevalence compared with those less disadvantaged in five of the seven states. In their study, disease burden was masked by low testing rates early in the pandemic especially in underserved areas, which may have obscured true associations. Our findings also indicated that pandemic stage likely contributed to differences in Baton Rouge and New Orleans.

To date, few studies have examined both area-level and individual-level factors’ influences on SARS-CoV-2 infection. After controlling for individual-level characteristics, Cromer [4] found that higher SARS-CoV-2 infection was associated with census-tract level factors, including higher population and household occupancy densities while percent of the population with a college degree was associated with lower infection risk. This differs from our findings in that our results were attenuated or not statistically significant once individual factors were included. This difference may be due to population or area-level variables (multiple census tract-level Census variables versus the zip code-level ADI composite variable) differences between the two studies.

The impact of the 17 components of ADI showed similar effect size magnitude and direction for infection risk across cities. We were unable to identify other studies which published associations for all the individual ADI components. However, previous studies reported similar associations for household crowding [1, 4, 16], unemployment [4], and household income [16], which suggests that they may be the most robust area-level predictors of SARS-CoV-2 infection warranting further study. Conflicting results were reported for poverty, home value, and education [4]. These differences may be due to how the ADI components were analyzed. Additionally, we recalculated ADI coefficients assuming that previously calculated coefficients would have changed following Hurricane Katrina. Surprisingly, they were not different than previously calculated coefficients.

Several individual factors independently contributed to infection risk in this study. After adjustment for ADI, race and age (Baton Rouge) and race, marital status, household size, and comorbidities (New Orleans) were significant. Older age is a well-known risk factor for SARS-CoV-2 infection and severe COVID-19 [17, 18] which contradicts our findings. Our finding that older age groups in Baton Rouge experienced lower infection risk is likely due to the stage of the pandemic during which data were collected. Black race has been noted as a risk factor for SARS-CoV-2 infection previously [1, 2, 4, 5, 7, 8, 19], and this study showed that self-reported race is a risk factor independent of ADI. Thus, further study of the underpinnings of SARS-CoV-2 infection racial disparities is needed.

Differences in community-level variability in infection and community- and individual-level associations in New Orleans compared to Baton Rouge may be due to the timing of each data collection period. In New Orleans (Spring 2020), little was known about the virus and public health messaging regarding masking, working from home, washing hands, and social distancing was new. Older age and comorbidities were not known risk factors for more severe disease. In contrast, Baton Rouge experienced their first large surge in Summer 2020, which represented a fundamentally different stage of the pandemic; the public was aware of risk factors for severe disease and state-level mask mandates and social distancing recommendations were in place. Importantly, businesses were closed, and stay-at-home orders were in place in New Orleans, but Baton Rouge was reopening during data collection. Thus, New Orleans communities may have been somewhat equally vulnerable to SARS-CoV-2 infection during this time whereas Baton Rouge communities with more access to health care, public health messaging, and resources to avoid infection likely decreased their risk of infection. For example, odds of infection were not different by age in New Orleans, but older individuals in Baton Rouge were less likely to be infected. Only the most essential employees worked on-site at the time of the New Orleans study, but many non-essential employees were back at work in Baton Rouge by July. Further, by this time, the risk of severe COVID-19 among older adults was well known which likely resulted in older adults actively decreasing their own risk by staying home, diligently wearing masks, etc. Further evaluation of how pandemic stage and public health recommendations affected disease risk is needed.

This study has several strengths and weaknesses that warrant discussion. The inclusion of information on area-level and individual-level risk factors coupled with seroprevalence data is a marked strength. Most previous studies have examined these issues using only area-level factors and their association with area-level infection rates. By including both, we showed that variables at both levels may contribute to higher infection odds with higher predictive power of individual compared to community characteristics. The use of representative samples from two municipalities at different stages of the pandemic is also a strength as previous studies compared areas during the same time frame. This allowed us to examine infection risk during and after stay-at-home orders, mask mandates, and the discovery of significant risk factors (i.e., age and certain co-morbidities). Finally, our ability to compute new ADI coefficients for Louisiana is an improvement over using existing coefficients derived using older data from different jurisdictions.

Our limitations include the use of ZCTAs rather than a more precise area measure, such as block group or census tract. A study of a Massachusetts hospital network found that although individual factors accounted for variation in infection risk, using census tract data enabled them to delineate more subtle area-level risk factors [4] versus a previous study that used cities or towns as areas [1]. Thus, with more granular area-level analysis (e.g. census tract versus ZCTA), we may have found additional area-level factors which increased infection risk. Additionally, ZCTA assignment based solely on participant zip code is imperfect [9]. In most instances an area’s zip code and ZCTA are the same or very similar [9]; however, this is not always the case. As such, misclassification bias related to spatial mismatch may be present. This may be especially true for New Orleans where low- and high-income and deprivation areas are often co-located within the same zip codes/ZCTAs. We were limited by incomplete address information on the participant questionnaire which only required five-digit home zip code. As such, the lack of valid home addresses precluded assignment of census tracts or block groups. Given the unique aspects of the present study, our use of ZCTAs to examine community-level SARS-CoV-2 risk, although imperfect, represents an important contribution to the literature and highlights future research needs to replicate these analyses using a lower level of geography. The degree to which our data at different levels are spatially correlated is not known, which emphasizes the observational nature of our design and analysis which further limits interpretations regarding causation in this study. In addition, ADI is only a single index of social determinants of health, and future studies could include additional, multidimensional indices [20] to clarify more nuanced risk factors for SARS-CoV-2 infection. Importantly, individual level variables accounted for in our models are intertwined with the ADI factors. For example, individuals’ household size, job type, and marital status are closely related to ADI variables household crowding, white-collar occupations, and single-parent households, respectively. However, analyses did not indicate issues of multi-collinearity or model instability. Finally, not all possible individual variables were accounted for in these analyses. Future studies should examine additional individual-level variables and multifaceted area-level measures (e.g. isolation) that may be associated with infection risk.

## Conclusions

While higher community-level deprivation was associated with higher odds of SARS-CoV-2 infection, individual-level characteristics accounted for most of the association. Data from two Louisiana seroprevalence studies at different stages of the SARS-CoV-2 pandemic show that ADI provided little additional information to explain infection risk after accounting for individual-level factors. Risk factors changed over time.

## Supporting information

S1 Table Relevant survey questions from prevalence studies. Questions were asked verbally and noted by research personnel. * indicates questions only asked in Baton Rouge.
(TIF)

S2 Table Area Deprivation Index (ADI) concepts with factor loading and coefficients calculated previously and in this study. (TIF)

S3 Table 2018 census data from Baton Rouge and New Orleans. Greater Baton Rouge includes the following parishes: Ascension, East Baton Rouge, Livingston, and West Baton Rouge. Greater New Orleans includes Jefferson and Orleans parishes. These were chosen to reflect the enrollment areas of the two seroprevalence studies.
(TIF)

S4 Table COVID-19 seroprevalence and ADI study data. All records used to generate this analysis are shown without identifying information.
(XLSX)

S1 Fig Unadjusted odds ratios and 95% confidence intervals of infection with severe acute respiratory syndrome 2 by ADI components per unit change in New Orleans and Baton Rouge, Louisiana. Blue indicates greater odds, yellow indicates decreased odds and black indicates no significant difference.
(TIF)
