# Supplementary figures and images for: Community versus individual risk of SARS-CoV-2 infection in two municipalities of Louisiana, USA: An assessment of Area Deprivation Index (ADI) paired with seroprevalence data over time
Source: PLoS One. 2021 Nov 30;16(11):e0260164. doi: 10.1371/journal.pone.0260164 (PMC8631658; doi:10.1371/journal.pone.0260164)

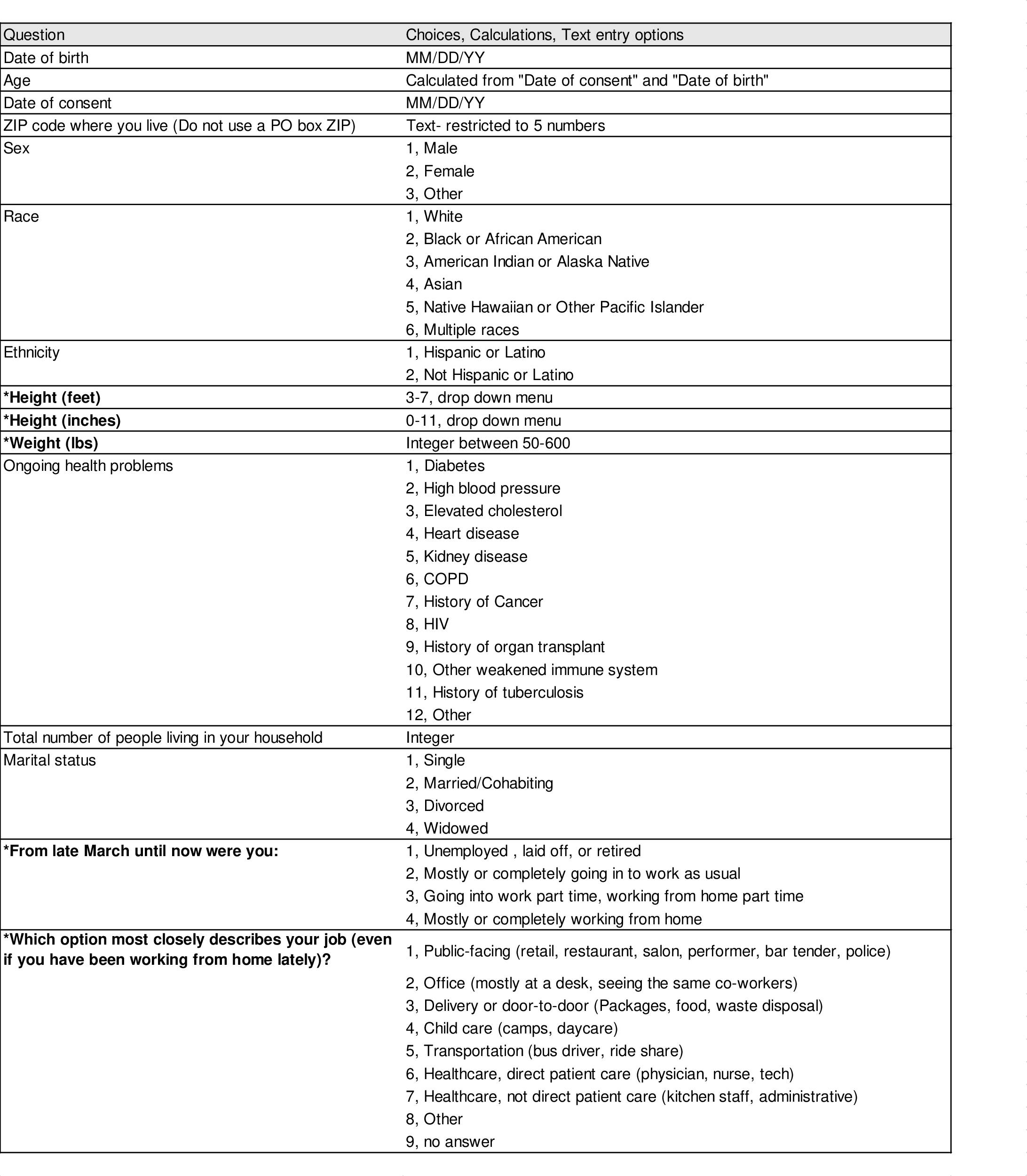

Supplement: S1 Table — Questions were asked verbally and noted by research personnel. * indicates questions only asked in Baton Rouge. (TIF) [file pone.0260164.s001.tif]

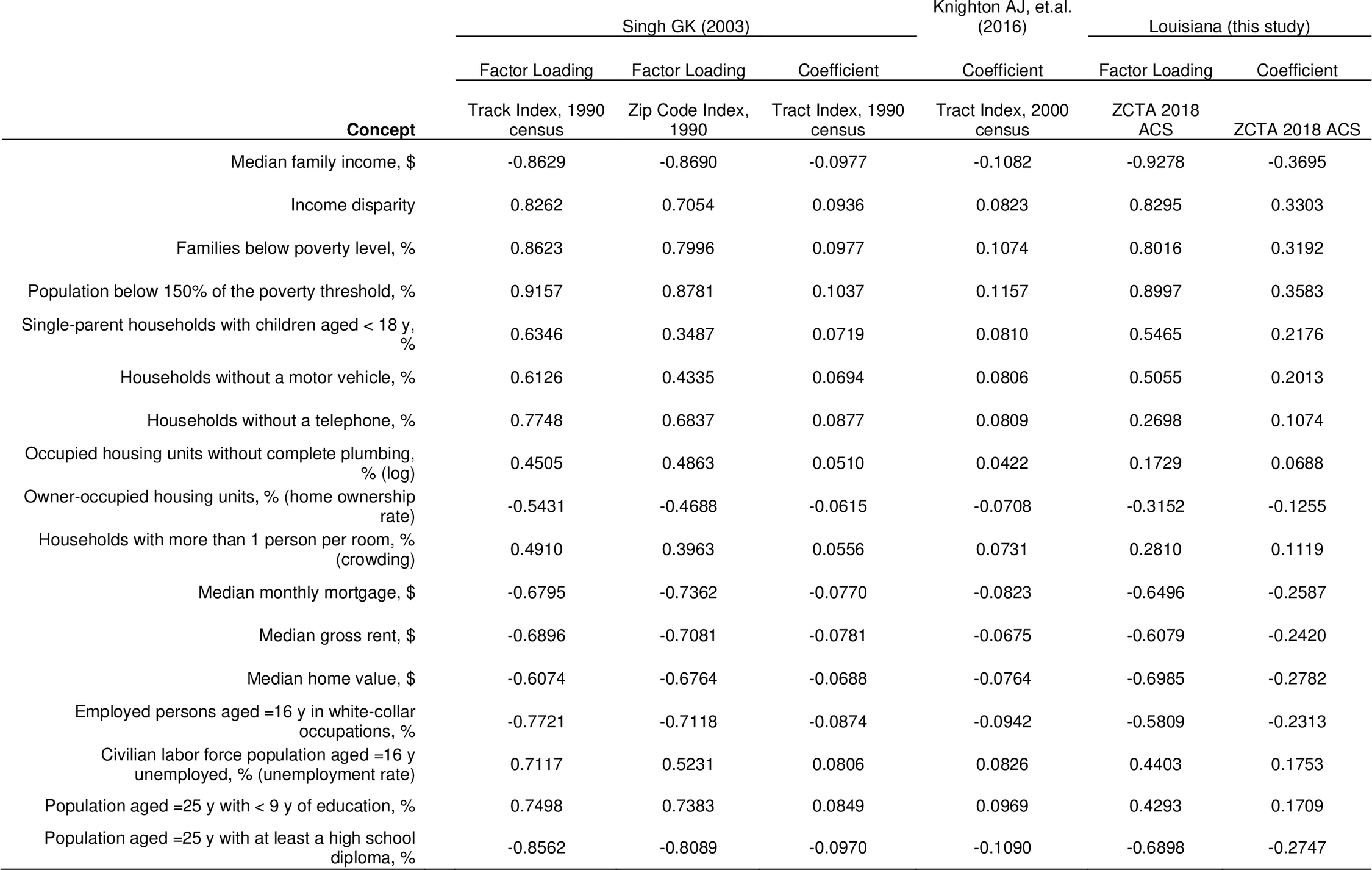

Supplement: S2 Table — (TIF) [file pone.0260164.s002.tif]

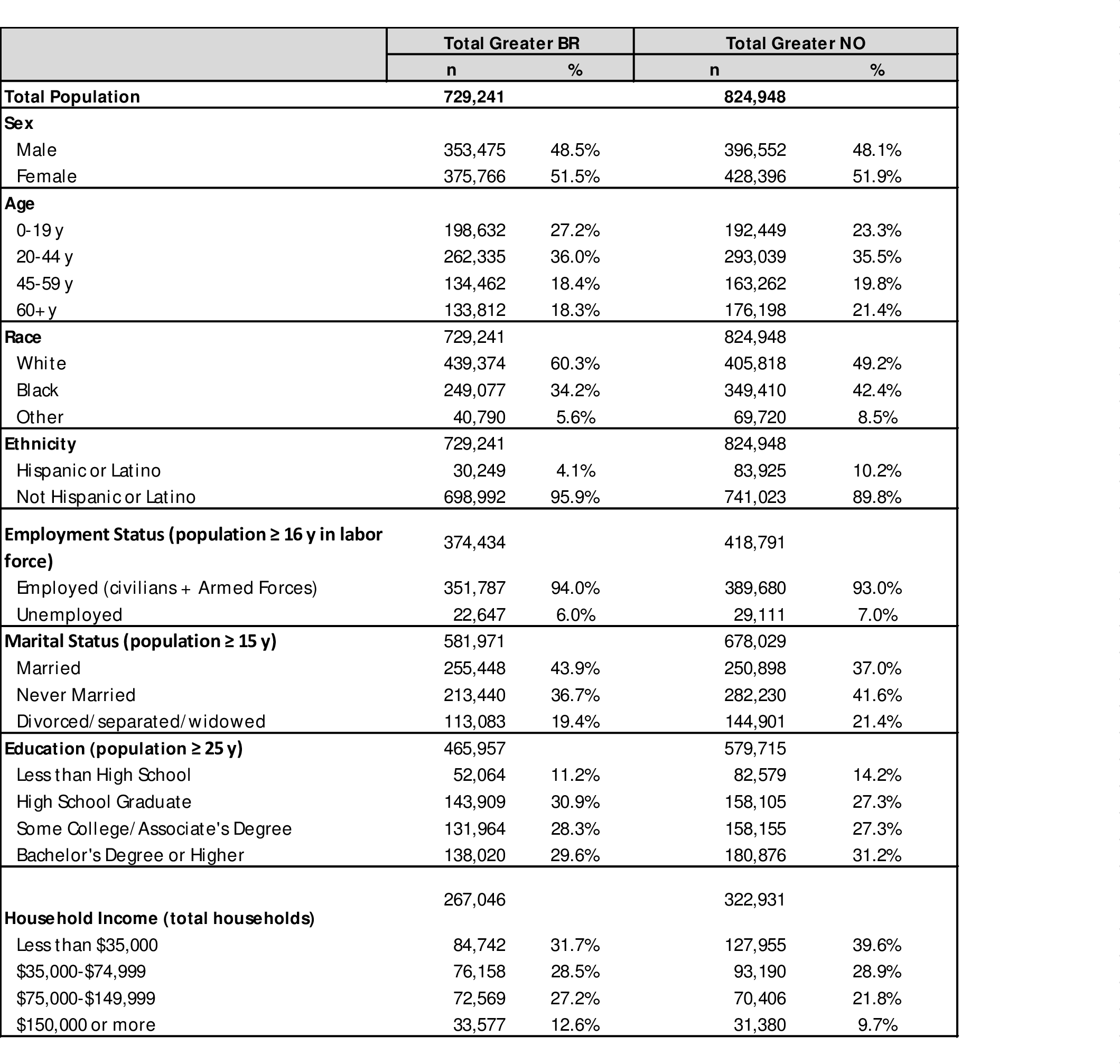

Supplement: S3 Table — Greater Baton Rouge includes the following parishes: Ascension, East Baton Rouge, Livingston, and West Baton Rouge. Greater New Orleans includes Jefferson and Orleans parishes. These were chosen to reflect the enrollment areas of the two seroprevalence studies. (TIF) [file pone.0260164.s003.tif]

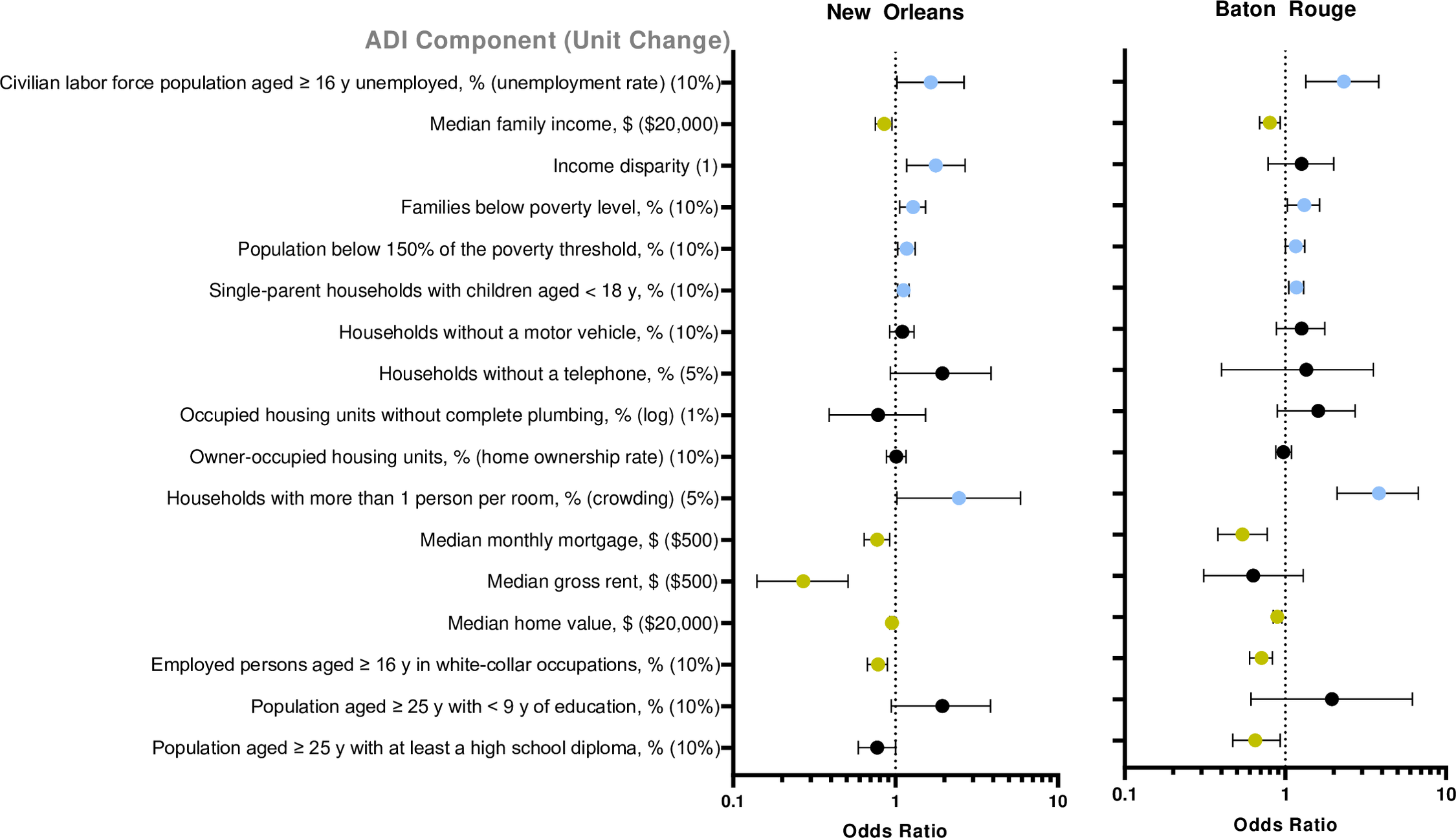

Supplement: S1 Fig — Blue indicates greater odds, yellow indicates decreased odds and black indicates no significant difference. (TIF) [file pone.0260164.s005.tif]
